# Supplementary material for: Linear epitope mapping in the E and NS1 proteins of dengue and Zika viruses: Prospection of peptides for vaccines and diagnostics
Source: PLoS One. 2023 Oct 3;18(10):e0292451. doi: 10.1371/journal.pone.0292451 (PMC10547212; doi:10.1371/journal.pone.0292451)
Supplement: S1 Checklist — (DOCX) [file pone.0292451.s001.docx]

STROBE Statement—checklist of items that should be included in reports of observational studies

|  | **Item No.** | **Recommendation** | **Page  No.** | **Relevant text from manuscript** |
| --- | --- | --- | --- | --- |
| **Title and abstract** | 1 | (*a*) Indicate the study’s design with a commonly used term in the title or the abstract | 2 | This cross-sectional study included individuals of all ages with laboratory confirmation of DENV and ZIKV infections. The participants were selected using convenience sampling. |
|  |  | (*b*) Provide in the abstract an informative and balanced summary of what was done and what was found | 2 | Dengue and Zika patients detected epitopes evenly distributed across the viral proteins in a peptide microarray platform. However, several epitopes were within “epitope hot spots” constituted by clusters of peptides recognized in more than 30% of the sub-arrays analyzed with individual or pooled serum samples. The serum samples of dengue and Zika patients showed a high level of cross-reaction for peptides in the DENV and ZIKV proteins. Analysis on an additional peptide microarray platform, which contained peptides selected based on the results of the first screening, revealed that two DENV peptides and one ZIKV peptide, highly-specific for their related viruses, were within the epitope hot spots; however, these peptides showed low detection rates (32.5, 35.0, and 28.6%, respectively). In addition, two DENV peptides detected at similarly high rates by dengue and Zika patients were also in the epitope hot spots. These hot spots contain several immunodominant epitopes that are recognized by a higher number of individuals when compared to the 15 amino acid sequence peptides. Thus, the entire epitope hot spots may have more potential than peptides of only 15 amino acids to serve as antigens in diagnostic tests and vaccine development. |
| **Introduction** | | | |  |
| Background/rationale | 2 | Explain the scientific background and rationale for the investigation being reported | 3-4 |  |
| Objectives | 3 | State specific objectives, including any prespecified hypotheses | 2 | We aimed to identify the linear epitope profile recognized by the serum samples of dengue and Zika patients in the E and NS1 proteins of DENV and ZIKV. |
| **Methods** | | | |  |
| Study design | 4 | Present key elements of study design early in the paper | 4-5 | This cross-sectional study, employing convenience sampling, was conducted from March 2017 to December 2022. The eligible population consisted of individuals of all ages with suspected dengue or Zika disease. Inclusion criteria required participants to have laboratory confirmation of DENV or ZIKV infection. Laboratory tests include the reverse transcription-polymerase chain reaction (RT-PCR), IgG/IgM tests, and/or plaque reduction neutralization test 50% (PRNT50). Exclusion criteria encompassed participants with negative IgG tests. The eligible control population comprised healthy individuals vaccinated against yellow fever, with negative serologic tests for dengue and Zika. Medical records served as the primary source of information regarding the presence of the disease, relying on laboratory test results. This assessment method served for the virus-infected groups and the healthy control group. |
| Setting | 5 | Describe the setting, locations, and relevant dates, including periods of recruitment, exposure, follow-up, and data collection | 4-5 | The Virology Research Center, School of Medicine of Ribeirao Preto, University of Sao Paulo, and the Virology Laboratory, School of Pharmaceutical Sciences, University of Sao Paulo were the centers for participant recruitment. All participants provided signed written consent for their inclusion in the study. When participants were children under 18 years old, parent/guardian consent was obtained, and the children themselves also signed the written assessment. In addition, archived serum samples from de-identified collections, dating from April 2011 to February 2020, located at the Virology Research Center, School of Medicine of Ribeirao Preto, University of Sao Paulo, the Virology Laboratory, School of Pharmaceutical Sciences, University of Sao Paulo, and the Production Department, Research Institute for Health Sciences, National University of Asuncion, Paraguay, were also included in the study. |
| Participants | 6 | (*a*) *Cohort study*—Give the eligibility criteria, and the sources and methods of selection of participants. Describe methods of follow-up  *Case-control study*—Give the eligibility criteria, and the sources and methods of case ascertainment and control selection. Give the rationale for the choice of cases and controls  *Cross-sectional study*—Give the eligibility criteria, and the sources and methods of selection of participants | 4 | The eligible population consisted of individuals of all ages with suspected dengue or Zika disease. Inclusion criteria required participants to have laboratory confirmation of DENV or ZIKV infection. Laboratory tests include the reverse transcription-polymerase chain reaction (RT-PCR), IgG/IgM tests, and plaque reduction neutralization test 50% (PRNT50). Exclusion criteria encompassed participants with negative IgG tests. The eligible control population comprised healthy individuals vaccinated against yellow fever, with negative serologic tests for dengue and Zika. |
|  |  | (*b*) *Cohort study*—For matched studies, give matching criteria and number of exposed and unexposed  *Case-control study*—For matched studies, give matching criteria and the number of controls per case |  |  |
| Variables | 7 | Clearly define all outcomes, exposures, predictors, potential confounders, and effect modifiers. Give diagnostic criteria, if applicable | 4 | Most of the countries in South America include the Yellow fever virus (YFV) vaccine in their vaccination calendars. YFV belongs to the Flavivirus genus, just like dengue and Zika viruses. This similarity can cause additional challenges in serological tests as cross-reactivity may arise during the diagnosis of dengue and Zika. |
| Data sources/ measurement | 8* | For each variable of interest, give sources of data and details of methods of assessment (measurement). Describe comparability of assessment methods if there is more than one group | 4-5 | Medical records served as the primary source of information regarding the presence of the disease, relying on laboratory test results. This assessment method served for the virus-infected groups and the healthy control group. |
| Bias | 9 | Describe any efforts to address potential sources of bias | 4 | The eligible control population comprised healthy individuals vaccinated against yellow fever, with negative serologic tests for dengue and Zika. |
| Study size | 10 | Explain how the study size was arrived at | 5 | The participants were selected using convenience sampling. Due to budget limitations, the number of available microarray slides determined the sample size. |

Continued on next page

| Quantitative variables | 11 | Explain how quantitative variables were handled in the analyses. If applicable, describe which groupings were chosen and why | 7 | Each spot median fluorescence intensity, with local background subtraction, was computed with the GenePix Pro 7 Molecular Devices, USA, or Mapix, Innopsys, France, software. Fluorescence intensity values <1 were converted to 0. Then, fluorescence intensity values were normalized against the negative controls (HA peptides). This normalization was done by dividing the fluorescence intensity value of each spot by the mean fluorescence intensity of the negative control spots. Normalized fluorescence intensity values <1 were converted to 1 and log2-transformed to reduce variability. The final fluorescence intensity value for each peptide was the mean of the fluorescence intensity value of the duplicated peptide spots. |
| --- | --- | --- | --- | --- |
| Statistical methods | 12 | (*a*) Describe all statistical methods, including those used to control for confounding | 7 and 8 | - The fluorescence intensity threshold for each peptide was the mean fluorescence intensity value plus two times the standard deviation of the mean intensity obtained with the control serum samples (24).  - We compared the peptide detection rates obtained with serum samples from dengue and Zika patients by constructing a contingency (2 × 2) table and analyzing the data using Fisher's exact test (25). All values were two-tailed, and the statistical significance threshold was p< 0.05. |
|  |  | (*b*) Describe any methods used to examine subgroups and interactions |  |  |
|  |  | (*c*) Explain how missing data were addressed |  |  |
|  |  | (*d*) *Cohort study*—If applicable, explain how loss to follow-up was addressed  *Case-control study*—If applicable, explain how matching of cases and controls was addressed  *Cross-sectional study*—If applicable, describe analytical methods taking account of sampling strategy |  |  |
|  |  | (*e*) Describe any sensitivity analyses |  |  |
| **Results** | | | | |
| Participants | 13* | (a) Report numbers of individuals at each stage of study—eg numbers potentially eligible, examined for eligibility, confirmed eligible, included in the study, completing follow-up, and analysed | 9 | This study included sera from 48 dengue patients, 28 Zika patients, and five healthy individuals. Healthy individuals with negative serologic tests for dengue and Zika and vaccinated against yellow fever served as controls. |
|  |  | (b) Give reasons for non-participation at each stage |  |  |
|  |  | (c) Consider use of a flow diagram |  |  |
| Descriptive data | 14* | (a) Give characteristics of study participants (eg demographic, clinical, social) and information on exposures and potential confounders |  |  |
|  |  | (b) Indicate number of participants with missing data for each variable of interest | 10 | Table 1 |
|  |  | (c) *Cohort study*—Summarise follow-up time (eg, average and total amount) |  |  |
| Outcome data | 15* | *Cohort study*—Report numbers of outcome events or summary measures over time |  |  |
|  |  | *Case-control study—*Report numbers in each exposure category, or summary measures of exposure |  |  |
|  |  | *Cross-sectional study—*Report numbers of outcome events or summary measures |  |  |
| Main results | 16 | (*a*) Give unadjusted estimates and, if applicable, confounder-adjusted estimates and their precision (eg, 95% confidence interval). Make clear which confounders were adjusted for and why they were included |  | - The fluorescence intensity threshold for each peptide was the mean fluorescence intensity value plus two times the standard deviation of the mean intensity obtained with the control serum samples (24). |
|  |  | (*b*) Report category boundaries when continuous variables were categorized |  |  |
|  |  | (*c*) If relevant, consider translating estimates of relative risk into absolute risk for a meaningful time period |  |  |

Continued on next page

| Other analyses | 17 | Report other analyses done—eg analyses of subgroups and interactions, and sensitivity analyses |  |  |
| --- | --- | --- | --- | --- |
| **Discussion** | | | | |
| Key results | 18 | Summarise key results with reference to study objectives | 19-24 |  |
| Limitations | 19 | Discuss limitations of the study, taking into account sources of potential bias or imprecision. Discuss both direction and magnitude of any potential bias | 24 | A limitation of our study was the low number of individuals in the control group. In regions endemic to dengue, such as Brazil and Paraguay, finding individuals not exposed to DENV is challenging. Despite this limitation, epitope hotspots identification within the viral proteins has provided valuable insights and instilled confidence in the results obtained from the study. The presence of these hot spots supports the notion that specific regions of the viral proteins play a crucial role in eliciting immune responses and can serve as potential targets for diagnostic tests and vaccine development. |
| Interpretation | 20 | Give a cautious overall interpretation of results considering objectives, limitations, multiplicity of analyses, results from similar studies, and other relevant evidence |  |  |
| Generalisability | 21 | Discuss the generalisability (external validity) of the study results |  |  |
| **Other information** | |  | | |
| Funding | 22 | Give the source of funding and the role of the funders for the present study and, if applicable, for the original study on which the present article is based |  |  |

*Give information separately for cases and controls in case-control studies and, if applicable, for exposed and unexposed groups in cohort and cross-sectional studies.

**Note:** An Explanation and Elaboration article discusses each checklist item and gives methodological background and published examples of transparent reporting. The STROBE checklist is best used in conjunction with this article (freely available on the Web sites of PLoS Medicine at http://www.plosmedicine.org/, Annals of Internal Medicine at http://www.annals.org/, and Epidemiology at http://www.epidem.com/). Information on the STROBE Initiative is available at www.strobe-statement.org.
