# Supplementary material for: Linear epitope mapping in the E and NS1 proteins of dengue and Zika viruses: Prospection of peptides for vaccines and diagnostics
Source: PLoS One. 2023 Oct 3;18(10):e0292451. doi: 10.1371/journal.pone.0292451 (PMC10547212; doi:10.1371/journal.pone.0292451)
Supplement: Attachment — Submitted filename: Response to Reviewers.docx [file pone.0292451.s007.docx]

**Answer to Editor and Reviewers**

**Editor**

1. Please ensure that your manuscript meets PLOS ONE's style requirements, including those for file naming.

**Answer**

The manuscript style was reviewed to meet PLOS ONE's style requirements.

2. We note that the grant information you provided in the ‘Funding Information’ and ‘Financial Disclosure’ sections do not match.

**Answer**

We have included the correct grant information: This study was financially supported by Fundação de Amparo à Pesquisa do Estado de São Paulo, FAPESP, Brazil (grant numbers 2017/09194-3 and 2019/26119-0), and Consejo Nacional de Ciencia y Tecnología, CONACYT, Paraguay (grant number PIRT19-1). The funders had no role in study design, data collection and analysis, decision to publish, or preparation of the manuscript.

3. Please upload a new copy of Figure 2 as the detail is not clear. Please follow the link for more information:

**Answer**

We uploaded an improved version of Figures 1 and 2.

4. We suggest you thoroughly copyedit your manuscript for language usage, spelling, and grammar. If you do not know anyone who can help you do this, you may wish to consider employing a professional scientific editing service.

**Answer**

The manuscript underwent professional copyediting by a scientific editing service, enhancing the overall linguistic quality.

**Reviewer #1:**

1- It would be helpful if the authors better described to the reader how to interpret Fig 1 and Table 2 together. Since the table and figure contain similar data, what is the purpose of including each? With a better explanation, it would make more sense to use both. Also, in Figure 1, it should be more clearly stated in the caption that the vertical bars represent patient samples. In Table 2, is the formatting is incorrect, or do some patients in the Zika population not have P and E data? There are blank rows.

**Answer**

We have enhanced the clarity of our manuscript by revising the text that elaborates on the findings presented in Fig 1 and Table 2 (lines 235-240 and 254-258). Additionally, we have provided in the caption a comprehensive explanation to enhance the interpretation of Fig 1 (lines 242-245).

The occurrence of empty rows within Table 2 resulted from initial formatting issues, which have been resolved.

2- For Table 7, it would be helpful if the authors highlighted rows with significant results or peptides for the reader to pay attention to.

**Answer**

We have highlighted the Percent Identity - Median (%) column values within Table 7, as they encompass the most pertinent results.

3- Table 4 and 6 – What is the purpose of including a column called “code?” This is not defined in the text.

**Answer**

Each peptide has a specific code within the microarray slide. However, we have removed the Code containing column from Tables 4 and 6 to avoid confusion.

4- Figure 2 is nearly illegible. Can the authors reduce the redundancy of text by combing cells that contain the same text? Also, what is the purpose of the “code” column? The figure needs to be simplified or split into multiple panels.

**Answer**

We uploaded an improved version of Figures 1 and 2.

5- Table 6 – why are the p-values such high numbers, greater than 1?

**Answer**

Apologies for the error. That specific column corresponds to the Detection rate (%) value, which has now been rectified.

6- Line 68 – “practically the entire country” is vague and colloquial. This should be more precise.

**Answer**

The manuscript underwent professional copyediting by a scientific editing service, enhancing the overall linguistic quality.

**Reviewer #2:**

1) Only 5 serum samples from YFV vaccinated and DENV/ZIKV seronegative healthy women were used as controls. From these, 4 or 3 serum samples were used for the epitope mapping or peptide microarray assays, respectively. To arrive to the conclusions the authors state in their work, a bigger and more representative (age-gender) population should be included as control group.

**Answer**

We agree with the reviewer's suggestion regarding the potential benefits of increasing the number of control samples to enhance the reliability of our study. As acknowledged in our manuscript, the limitation of our study lies in the constrained availability of resources, preventing us from conducting additional microarray assays. Nonetheless, we maintain confidence in the presented results. We have revised the discussion section to incorporate relevant findings from the literature that further support our conclusions (lines 428-441).

2) It is not clear to me how many DENV serum samples, from each serotype, have been used in the work. Authors should characterize and inform the number of samples collected and analyzed for each DENV serotype, in order to demonstrate similar sample sizes for each of the four DENV serotypes.

**Answer**

The information regarding the DENV serotype at the time of recruitment has been incorporated into Table 1. However, it's important to note that we did not include an analysis of this variable in our study. This decision stems from the fact that in countries with DENV transmission, such as Brazil and Paraguay, individuals often encounter multiple DENV serotype infections throughout their lives. The identification of these infections can be a formidable challenge. The widespread prevalence of multi-serotype infections introduces a significant layer of complexity when attempting to explore potential associations between DENV epitope detection and the specific DENV serotype individuals had at the time of recruitment.

3) How do you establish the 30% threshold detection rate value for the determination of hot spots?

**Answer**

We rephrase the text for clarification (lines 262-264).

"When analyzing peptide detection rates, we identified epitope hotspots constituted by clusters of peptides that consistently exceeded a 30% detection rate".

4) Line 36-37: Rephrase "Dengue and Zika patients detected..."

**Answer**

The manuscript underwent professional copyediting by a scientific editing service, enhancing the overall linguistic quality.

5) Line 148: "Dyligt" misspelt

**Answer**

The spelling of "Dyligt" was corrected.

6) Line 215-216: Correct "The average age was 33 and 34.4 years for dengue Zika..."

**Answer**

The statement was corrected (lines 219-220).

7) Table 6: "p-value" column shoud be "Detection rate (%)" instead

**Answer**

We apologize for the error that occurred. We have rectified the issue.

8) Line 348: "ZKIKV" misspelt

**Answer**

The spelling of "ZKIKV" was corrected.

9) The Discussion section is too long. It should be shortened.

Since the PLOS ONE journal does not impose restrictions on manuscript length, we respectfully seek the reviewer's approval to maintain the expanded Discussion section. We have revised this section to improve its overall clarity. Furthermore, we have included an additional statement to strengthen our findings and acknowledge the study's limitation arising from the limited number of control samples as mentioned above.
